# Assessing complexity and dynamics in epidemics: geographical barriers and facilitators of foot-and-mouth disease dissemination

**DOI:** 10.3389/fvets.2023.1149460

**Published:** 2023-05-12

**Authors:** A. L. Hoogesteyn, A. L. Rivas, S. D. Smith, F. O. Fasina, J. M. Fair, M. Kosoy

**Affiliations:** ^1^Department of Human Ecology, CINVESTAV, Merida, Yucatan, Mexico; ^2^Center for Global Health, Internal Medicine, School of Medicine, University of New Mexico, Albuquerque, NM, United States; ^3^Geospatial Research Services, Ithaca, NY, United States; ^4^Department of Veterinary Tropical Diseases, Faculty of Veterinary Science, University of Pretoria, Pretoria, South Africa; ^5^ECTAD Food and Agriculture Organization (FAO), Nairobi, Kenya; ^6^Bioscience Division, Los Alamos National Laboratory, Los Alamos, NM, United States; ^7^KB One Health LLC, Fort Collins, CO, United States

**Keywords:** medical geography, complexity analysis, emergence, epidemics, foot-and-mouth disease, movement ecology

## Abstract

**Introduction:**

Physical and non-physical processes that occur in nature may influence biological processes, such as dissemination of infectious diseases. However, such processes may be hard to detect when they are complex systems. Because complexity is a dynamic and non-linear interaction among numerous elements and structural levels in which specific effects are not necessarily linked to any one specific element, cause-effect connections are rarely or poorly observed.

**Methods:**

To test this hypothesis, the complex and dynamic properties of geo-biological data were explored with high-resolution epidemiological data collected in the 2001 Uruguayan foot-and-mouth disease (FMD) epizootic that mainly affected cattle. County-level data on cases, farm density, road density, river density, and the ratio of road (or river) length/county perimeter were analyzed with an open-ended procedure that identified geographical clustering in the first 11 epidemic weeks. Two questions were asked: (i) do geo-referenced epidemiologic data display complex properties? and (ii) can such properties facilitate or prevent disease dissemination?

**Results:**

Emergent patterns were detected when complex data structures were analyzed, which were not observed when variables were assessed individually. Complex properties–including data circularity–were demonstrated. The emergent patterns helped identify 11 counties as ‘disseminators’ or ‘facilitators’ (F) and 264 counties as ‘barriers’ (B) of epidemic spread. In the early epidemic phase, F and B counties differed in terms of road density and FMD case density. Focusing on non-biological, geographical data, a second analysis indicated that complex relationships may identify B-like counties even before epidemics occur.

**Discussion:**

Geographical barriers and/or promoters of disease dispersal may precede the introduction of emerging pathogens. If corroborated, the analysis of geo-referenced complexity may support anticipatory epidemiological policies.

## Introduction

To occur, epidemics involve more than a pathogen and a susceptible group of hosts. In addition to immunological, microbiological and demographic factors, numerous factors (including but not limited to the geographical environment) may also influence the development and progression of epidemics. To explore such factors, the analysis of variables that can be defined in terms of geographical coordinates (geo-referenced variables) has been proposed (1–5).

Numerous calls have suggested the development of methods that address complexity and dynamics in epidemiology (6, 7). Given its potential relevance in prevention, the study of disease dissemination with geo-referenced data is a topic of particular interest (8).

While some geographical factors (such as the road structure) may facilitate disease dispersal, other factors may act as barriers (1, 9). Yet, geographical factors do not have a single and constant role–they change over time and/or across space. For instance, both low and high road density may prevent disease dissemination. While low road density tends to prevent disease dispersal, high road density may also act as a barrier because, in highly urbanized areas (where road density is invariably high), roads compete against farming for land and, consequently, high road density may inadvertently block dissemination of infections affecting domestic animals (10).

Similarly, bridges may play different roles (11). If used to control epidemics (e.g., as disinfection sites), bridges act as obstacles, complementing the natural barrier effect exerted by rivers and other geographical features, such as mountains. However, in their typical usage—connecting regions separated by rivers—bridges may foster epidemic spread. Consequently, the study of geographical facilitators or non-facilitators of epidemic dissemination is not a discrete and/or static endeavor: it involves the analysis of dynamic interactions among pathogens, hosts, and geography. Rivers on the other hand most likely act as barriers for animal movement, and therefore, prevent infectious diseases. For example, rivers are suggested to shape present-day patterns of ecological and genetic variation among Amazonian species and communities (12).

To study dynamics (interactions that change over time), complexity should be considered. Measuring complexity is not a trivial endeavor because the mathematics (if not also the biology) influencing one scale may differ from the factors that affect other scales (13).

To investigate epidemics, “information” (not just “data”) should be generated. Data analysis is not enough–data structuring is needed (14). Structured data may reveal informative data patterns not directly conveyed by simple (non-structured) data.

Data structuring that focuses on *relationships* is not common (15). Much less so is the analysis of dynamic and complex relationships that include but exceed medical expertise (16). The information generated by structured data also depends on the data *format* utilized: for instance, it is not the same to read numbers from a table that lacks relationships than to directly visualize 3D patterns on a map (17–19). The validity and/or informative value of structured data can be objectively determined: it only requires determining whether structured data (complex indicators that include multiple variables) inform more than non-structured (simple) variables (20).

Because ‘point predictions’ (e.g., the specific number of a specific variable that differentiates two or more specific conditions at a specific time and place) depend on highly variable initial conditions, complexity analysis does not attempt to make long-term predictions. Instead, it focuses on properties (21). Complex systems possess at least three properties: (i) emergence, (ii) irreducibility, and (iii) unpredictability (21–23). *Emergence* (also known as *novelty*) refers to the fact that complex systems are multi-level structures, which reveal *new* features or functions only when the most complex (system-level) structure is assembled. *Irreducibility* means that *emergence* cannot be shown by or reduced to the properties of any one ‘simple’ (non-structured or low-level) variable. *Unpredictability* refers to the inability to predict emergence when only ‘simple’ and/or isolated variables are analyzed.

While investigated in infectious diseases, complexity and dynamics have been poorly explored in geo-referenced studies of epidemic dispersal. Yet, several properties of biological complex and dynamic systems are already well known in infectious diseases (24). For example*, data circularity* (data with no beginning and no end) is the essence of *seasonality*—one factor known to influence geo-epidemiology (25). Detecting such properties in epidemics matters because, given the highly combinatorial nature of complex and dynamic systems, numerous informative patterns may be embedded in the data, which may be missed by simple approaches (12, 26). If properties that characterize dynamic complexity were demonstrated in geo-referenced epidemics, it could then be explored whether some geographical factors may act as facilitators or barriers of epidemics.

To that end, data previously analyzed are here re-investigated (10). The reason to re-assess data collected in the 2001 Uruguayan FMD epizootic is because it predominantly affected bovines (a species that displays observable signs when infected by the FMD virus) and, at the time, all bovines in Uruguay were susceptible (no vaccine against FMD had been used in the previous decade). While other (non-bovine) species do not always reveal clinical signs when affected by the FMD virus (27), both the geographical location of the onset and the geo-temporal progression of the 2001 Uruguayan FMD epizootic were unambiguously recorded. While the purpose of this study is not to explore how FMD epizootics can disseminate or how the 2001 Uruguayan episode took place [such questions have been addressed in numerous, earlier studies (10, 28, 29)], data collected in that epizootic are used to ask two questions: (i) do epidemics reveal properties typical of complex systems? and (ii) if so demonstrated, could such properties distinguish geographical factors that may act as facilitators or barriers of epidemic spread?

## Materials and methods

### Materials

Two hundred and seventy-five counties of Uruguay were investigated. County-level geographical variables were combined with epidemic data collected in the first 11 weeks of the 2001 FMD Uruguayan epidemic (10, 29). These data were complemented with non-epidemiologic, geo-referenced data on area- and line-based structures (counties, rivers and roads, as reported in https://srvgisportal.igm.gub.uy/portal/apps/webappviewer/index.html?id=26d59683d5cb475fa70e8223fa0da173; Supplementary Table 1). Seven variables were investigated: county area (sq. km), FMD case density (infected farms/sq. km), farm density (farms/sq. km), road density (km of county road length/county area), river density (km of river length/county area), and the percent of county perimeter occupied by roads or rivers (road [river] length/county perimeter).

### Method

An open-ended, combinatorial approach was used, which investigated georeferenced and/or epidemiologic variables until visually distinct data patterns were detected in the early epidemic phase (first 2 weeks). Such patterns were then used to classify counties into two categories: facilitator [F] or barrier [B] of epidemic dispersal. Subsequent data analyses focused on detecting properties associated with complex and dynamic systems, such as emergence, irreducibility, and/or unpredictability. The method operated as an open-ended series of multivariate, map-based analyses which, after comparing several data ranges of each variable, concluded when at least one distinct pattern of geographical units (counties) was identified. Such a pattern should include not more than one dissimilar unit into a cluster of units otherwise similar, e.g., a cluster of was characterized by counties with similar values of the same geographical feature or not more than one county exhibiting dissimilar values.

Using a commercial Geographical Information Systems (*ArcGIS 9.3*, ESRI, Redlands, CA, United States) package, a layer (shapefile) of county boundaries was created. The seven variables used in the analysis were generated from variable layers and the county boundaries layer utilizing buffer, intersection and other data manipulation tools. The resulting data for these seven variables were combined into a single table which was then joined (appended) to the county boundaries layer using the common county identifier. The resulting dataset was then used to conduct a *query* for counties that fell within specified intervals (e.g., “road density greater than … AND river length segments in perimeter less than …”), and a *new set* was created. Such procedure was conducted for the entire 11-week long epidemics and for each epidemic week. The corresponding tables were then exported to a commercial statistical package (*Minitab 22*, Minitab Inc., State College, PA, United States) for further analyses. Correlation analysis was used to explore simple relationships among variables. The Mann–Whitney test for comparison of medians was applied to compare groups of counties. The same package was utilized to generate three-dimensional plots. A proprietary algorithm was used to facilitate the open-ended cycle that included map-based and 3D plot-based assessments.

## Results

**Figure 1:**
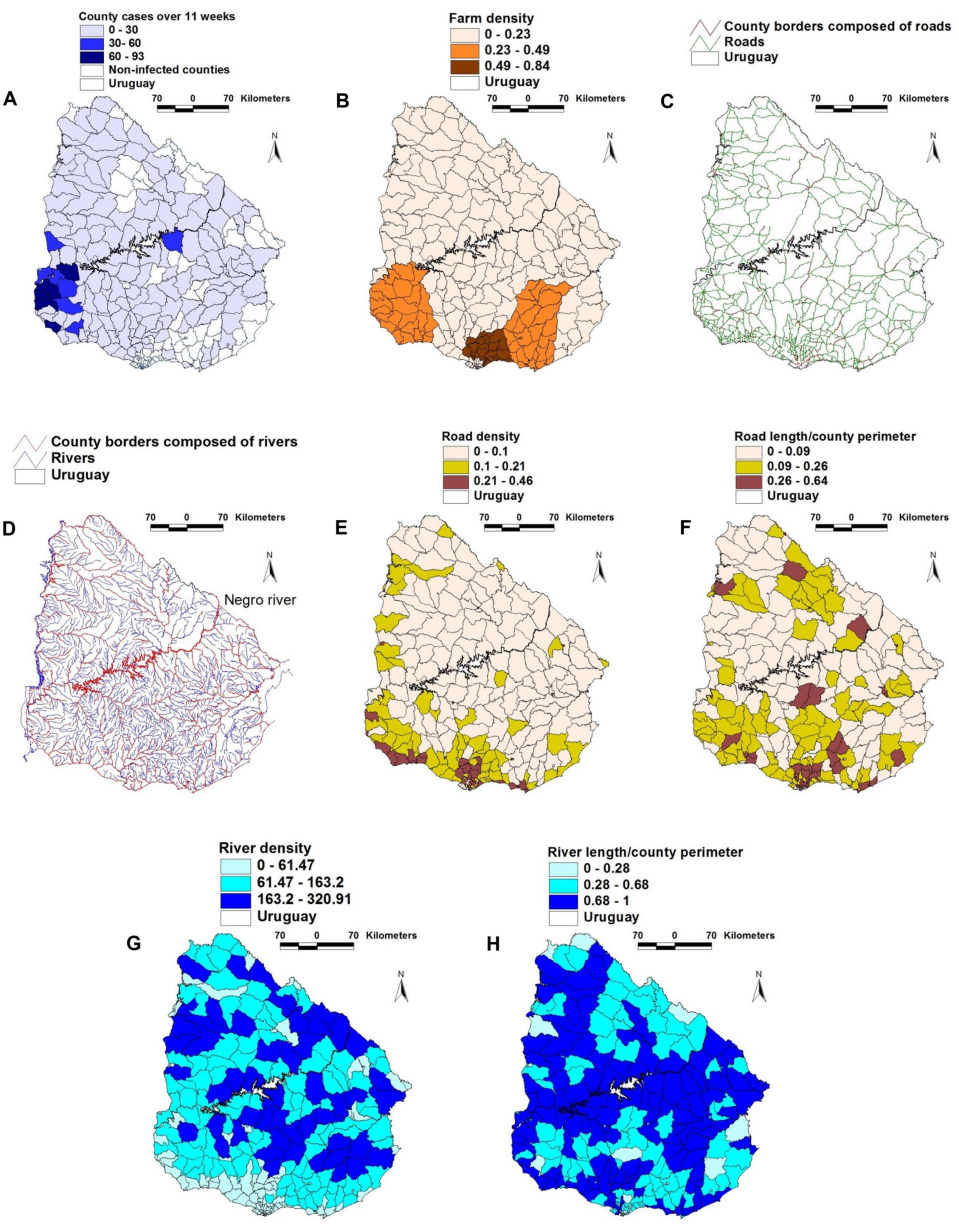
Geographical features of the 2001 FMD epizootic that took place in Uruguay. **(A)** county-wide FMD case density (cases/sq. km) reported in the first 11 weeks of the epizootic. **(B)** county farm density (farms/sq. km). **(C)** road and county boundaries composed of roads **(D)** rivers and county boundaries composed of rivers. **(E)** county road density. **(F)** county road length/county perimeter composed of roads. **(G)** county river density. **(H)** county river length/county perimeter composed of rivers. County case density was higher in the south-western region **(A)**, south of the Negro river, which flows diagonally trough the country, from the north-eastern to the south-western border **(D)**. The south-western region, as well as much of the southern coast shows the highest road density **(F)**. Such geo-epidemic structure suggests that the Negro river acted as a *de facto* obstacle for the dissemination of the epidemic, which was first reported to the south of this river.

The geographical location of FMD cases, farms, rivers, and roads is shown in Figures 1A–H. A physical barrier was observed: most FMD cases were located south of the Negro river (Figure 1D).

When geographical variables were analyzed (without considering temporal-epidemiologic data), *farm density* was positively associated with both *road density* and *road length* (both with *r* ≥ 0.34, *p* < 0.01). In contrast, *road length* was negatively and statistically significantly associated with *river length* (Supplementary Table 2). When epidemic and temporal data were assessed, positive and significant correlations were found between *case density* and both *farm density* and *road density* in at least one of the first two epidemic weeks. In the early epidemic phase, *farm density* was positively and significantly correlated with *road length* –a variable associated with *road density* (Supplementary Table 3). The values of the geographic variables changed over time: while infections were only reported in 29 of the 275 counties in epidemic week I, 71 counties reported FMD cases 1 week later (Supplementary Table 4A).

**Figure 2:**
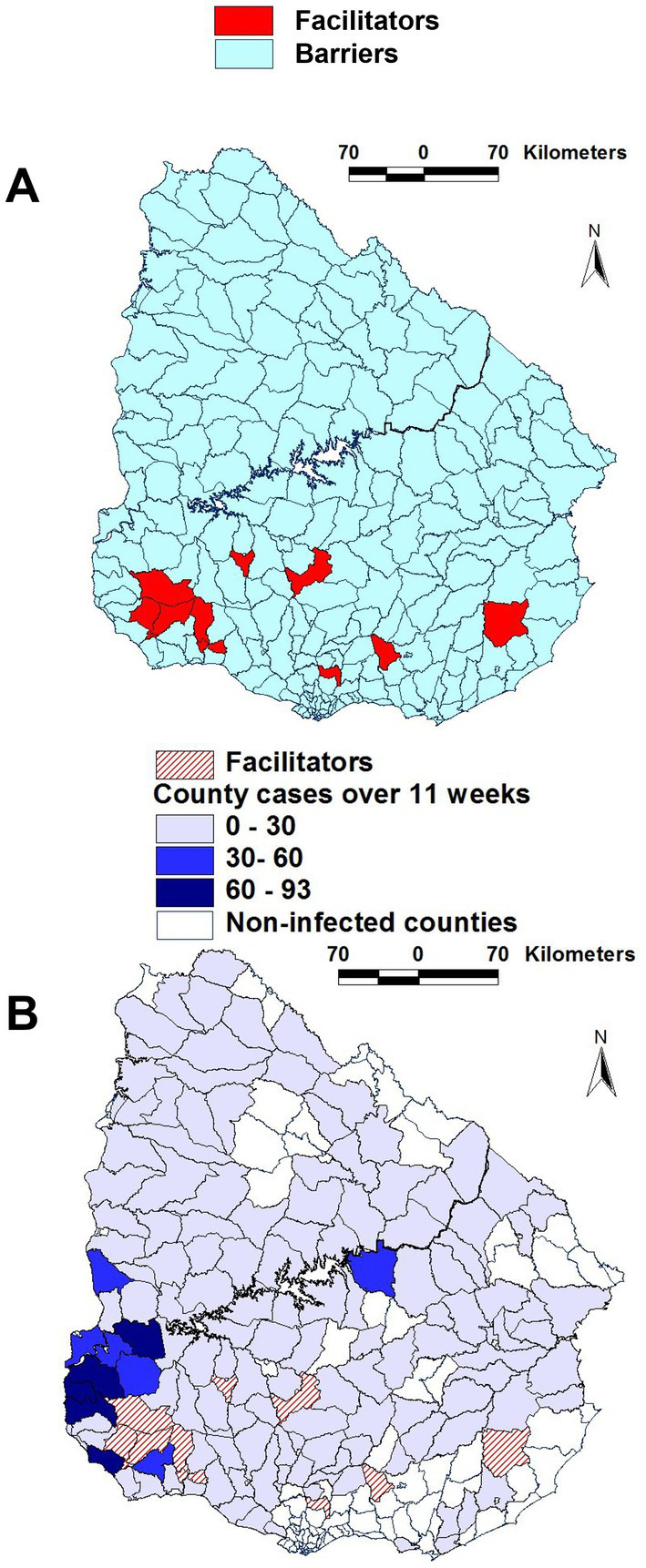
Geographic features of counties suspected to facilitate or block epidemic dispersal. Most ‘facilitator’ counties were clustered in the south-western region, where they occupied a continuous area **(A)**. To elucidate whether such pattern was due to chance or geo-biology, time-related epidemic data were investigated. Most ‘facilitator’ counties were contiguous to a group of counties where the highest case density was reported over the entire course of the epizootic **(B)**.

While correlation analysis indicated relationships, it did not distinguish county categories. In contrast, map-based assessments of complex data combinations detected a group of 11 counties here labeled as ‘facilitators of epidemic spread’ (F). Most ‘facilitator’ (F) counties clustered in the south-western region of the country (Figure 2A). ‘Facilitator’ counties were characterized by: (i) *farm density* > 0.15; (ii) *road density* > 0.1; (iii) *river density* > 0.1 but <0.3; (iv) *road length / county perimeter* > 0.1; and (v) *river length/county perimeter* > 0.38 but <0.6. The remaining 264 counties were classified as ‘barriers’ (B, Figure 2A).

To validate such a classification, FMD case data were assessed over time in F and B counties. Over 11 epidemic weeks, 130 and 1,420 cases were reported in F and B counties, respectively (Supplementary Table 4B). That is, the percentage of all cases associated with ‘facilitator’ counties (8.4% or 130/1550) was 2.1 times higher than the percent of cases within all counties (4.0% or 11/275). The F cluster was also geographically connected: it showed a continuous and contiguous structure within which, over 11 weeks, the highest FMD case density was observed (Figure 2B).

**Figure 3:**
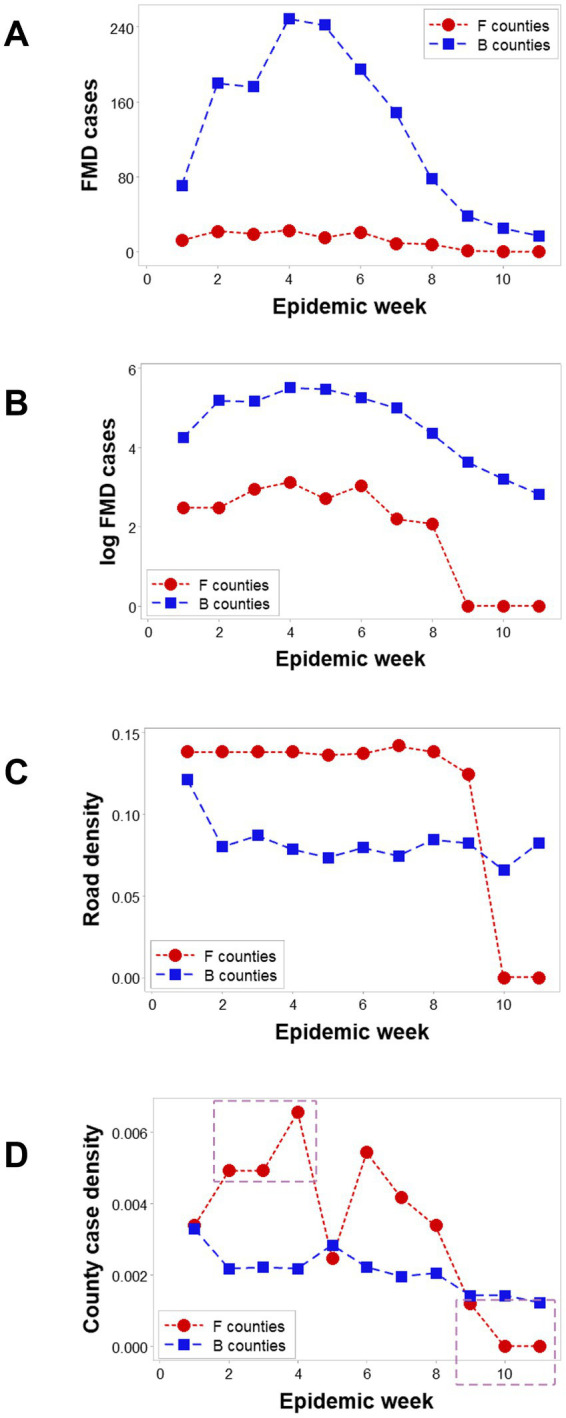
Dynamics of counties suspected to facilitate or block disease dispersal. In all 11 epizootic weeks, ‘barrrier’ (B) counties reported FMD cases; in contrast ‘facilitator’ (F) counties only presented cases in the first 9 weeks **(A)**. Log-transformed FMD case data revealed that F and B counties revealed similar trends in the first 9 weeks **(B)**. The median road density (km of road length/sq.km of county area) was significantly higher in F than B counties in the first 9 weeks **(C)**. FMD case density was higher in F than B counties in three of the first 4 weeks (oval, **D**), becoming zero after week 8 (box, **D**).

Spatial epidemic dynamics differed markedly between F and B counties. While FMD cases were reported in B counties in every week, no infection took place in F counties at weeks 10 and 11 (Figures 3A,B). The median *road density* associated with F counties was higher than that of B counties in the first 9 epidemic weeks (*p* < 0.01, Mann–Whitney test; Figure 3C). Epidemic dynamics also differed between county classes: the *case density* (cases/sq. km) was higher in F than B counties in epidemic weeks 2–4, but lower, later (rectangles; Figure 3D).

**Figure 4:**
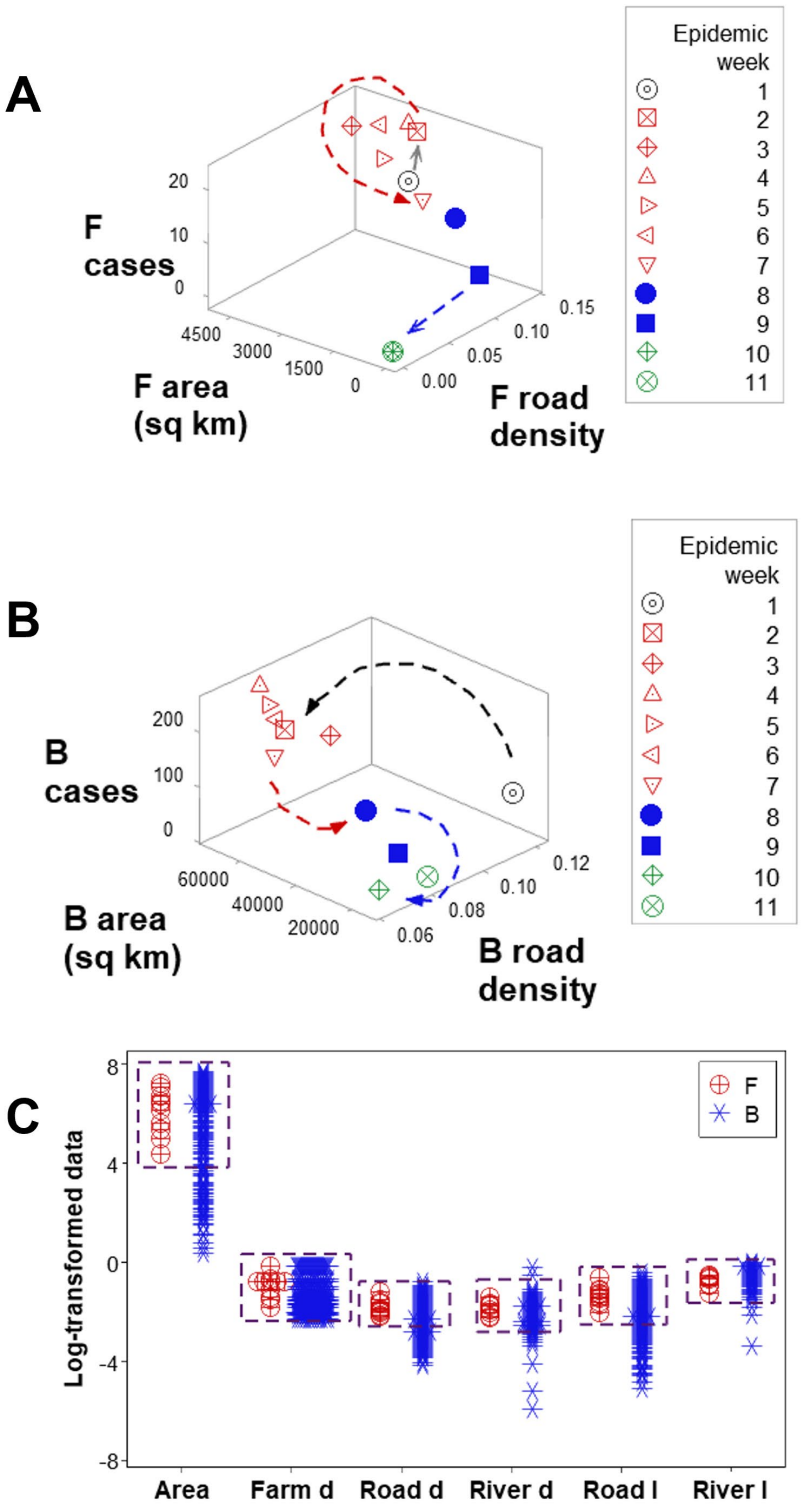
Complex and simple data assessments When F counties were considered, three epidemic phases were detected (arrows, **A**). When county classes were not considered (F and B classes were not differentiated), only two epidemic phases were differentiated (arrows, **B**). While three-dimensional, complex assessments distinguished F from B counties, no variable, alone, differentiated F from B counties: overlapping data distributions were observed (boxes, **C**).

Three-dimensional (3D) analyses revealed three temporal data inflections when the *road density* associated with F counties was measured together with the weekly *case count* and the *area* (sq km) of such counties (Figure 4A). Such indicators differentiated three epidemic phases, here described as early, intermediate, or late (or resolution; red, blue, and green symbols; Figure 4A). However, when such variables were assessed in B counties, only two data inflections were detected, and the last epidemic phase (resolution) was not observed (Figure 4B). Hence, a new (*emergent*) pattern (three, as opposed to two data patterns) was only displayed by F counties. In contrast, unstructured data, alone, did not distinguish F from B counties (Figure 4C). Because *emergence* was not predicted by or reduced to the properties of any one unstructured variable, the data demonstrated three typical properties of biological complexity: *emergence, irreducibility* and *unpredictability*.

**Figure 5:**
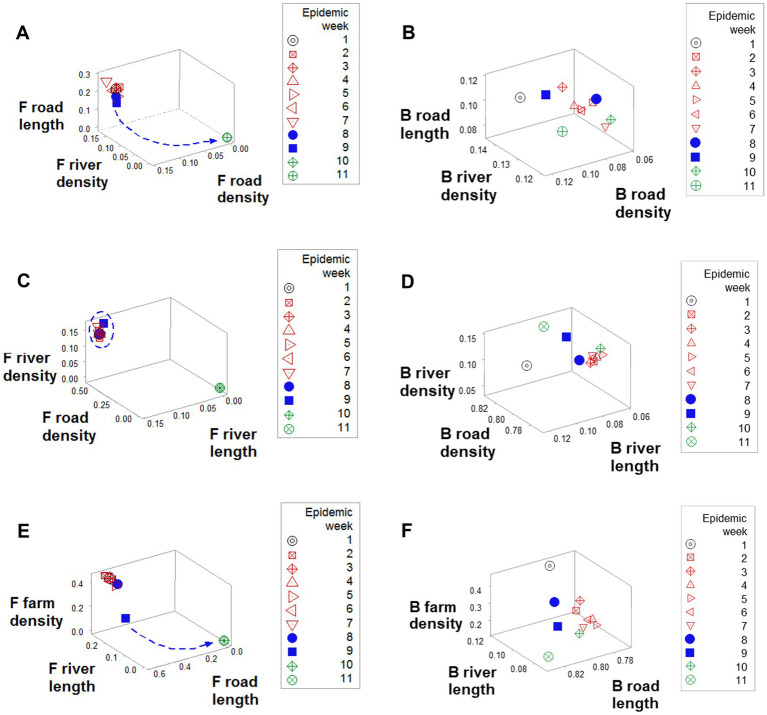
Geographical differences between F and B counties. Data clustering, as well as a perpendicular data inflection (shown by F counties after epidemic week 8), **(A)** were not observed in B counties **(B)**. While F counties displayed a high road density in the first 9 epidemics (circle, **C**), in B counties road density was high only in week 1 (arrow, **D**). F road length data revealed a sudden decrease data that predicted resolution (arrow, **E**), which was not shown the road length of B counties **(F)**.

*Emergence* was also documented when *road length*, *road density*, and *river density* were considered: a perpendicular data inflection, observed in F counties (arrow, Figure 5A), was not revealed by B counties (Figure 5B). While F counties exhibited a high *river density* only in the first epidemic week (oval, Figure 5C), B counties displayed a high river density throughout the first 9 epidemic weeks (Figure 5D). While, in F counties, a sudden decrease in *road length* values predicted resolution (blue arrow; Figure 5E), B counties did not express such a feature (Figure 5F).

**Figure 6:**
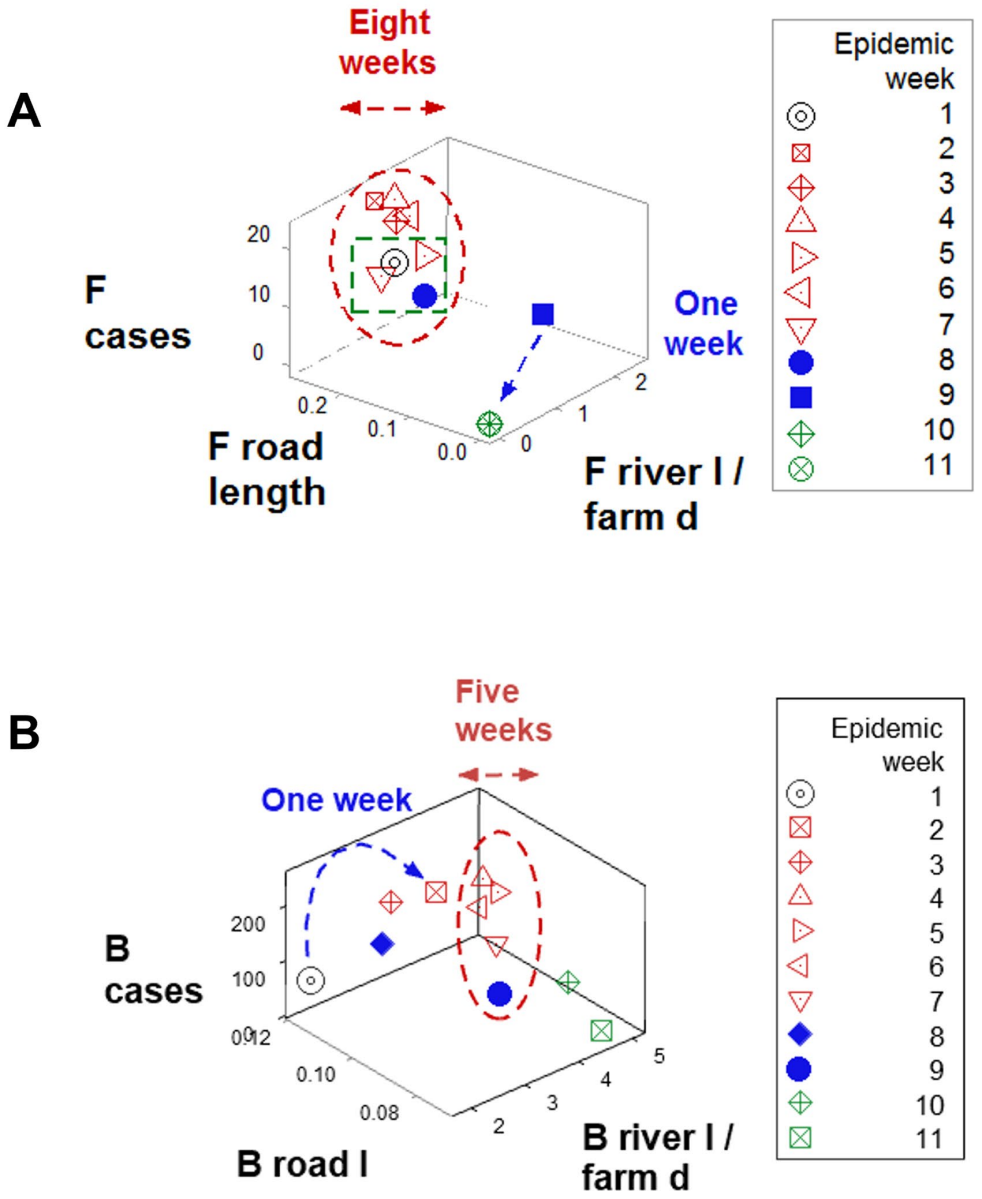
Demonstration of spatial–temporal biological relativity (ambiguity). **(A)** Oscillatory or circular data patterns were observed in numerous plots, including this one. One associated property is spatial–temporal biological relativity. Such a property is expressed as data points that describe long temporal periods but occupy a small space of the plot (red oval, **A,B**), as well as the opposite pattern: data points collected over a short time period, which occupy a large portion of space (blue lines, **A,B**). For instance, observations collected over 6 weeks (red oval, **B**) occupied a smaller space than observations collected over 1 week (blue line, **B**). This property results in ambiguity: some data points of similar values in all variables may possess different meaning, e.g., the open (red) symbols refer to the early epidemic stage, while the closed (blue) symbols reflect the resolution phase (green boxes, **A,B**).

Findings also revealed data *circularity* and *spatial–temporal biological relativity*. Circularity was shown in numerous expressions (Figures 4A,B and also in Figures 6A,B). Relativity was expressed both as data points that occupied a large portion of the space analyzed but involved a small period of time (blue lines; Figures 6A,B) and also as observations generated over a long period of time, which occupied a small plot space (red ovals; Figures 6A,B).

**Figure 7:**
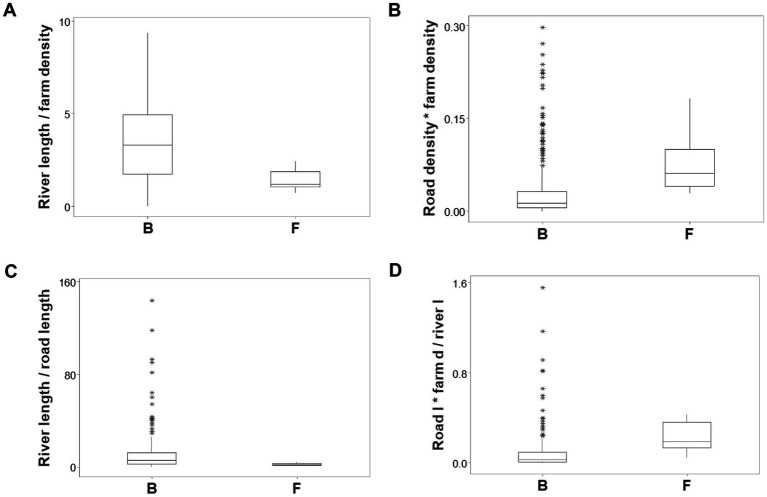
Bi-dimensional analysis of geo-referenced, complex physical relationships that may influence disease dispersal. Dimensionless indicators that included ratios and/or products showed statistically significantly different medians when F and B counties were compared (all at *p* ≤ 0.01, Mann–Whitney test, **A–D**). **(A)** River length/farm density (the ratio resulting from dividing river length [percent of county perimeter] over farm density). **(B)** Road density * farm density (the product resulting from multiplying road density times farm density). **(C)** River length/road length (the ratio resulting from dividing river length [percent of county perimeter] over road length [percent of county perimeter]). **(D)** [Road density/river density] * farm density (the result from dividing road density over river density, multiplied by farm density). After two products were calculated (river density times river length, and road density times farm density), the first product was divided by the second product.

**Figure 8:**
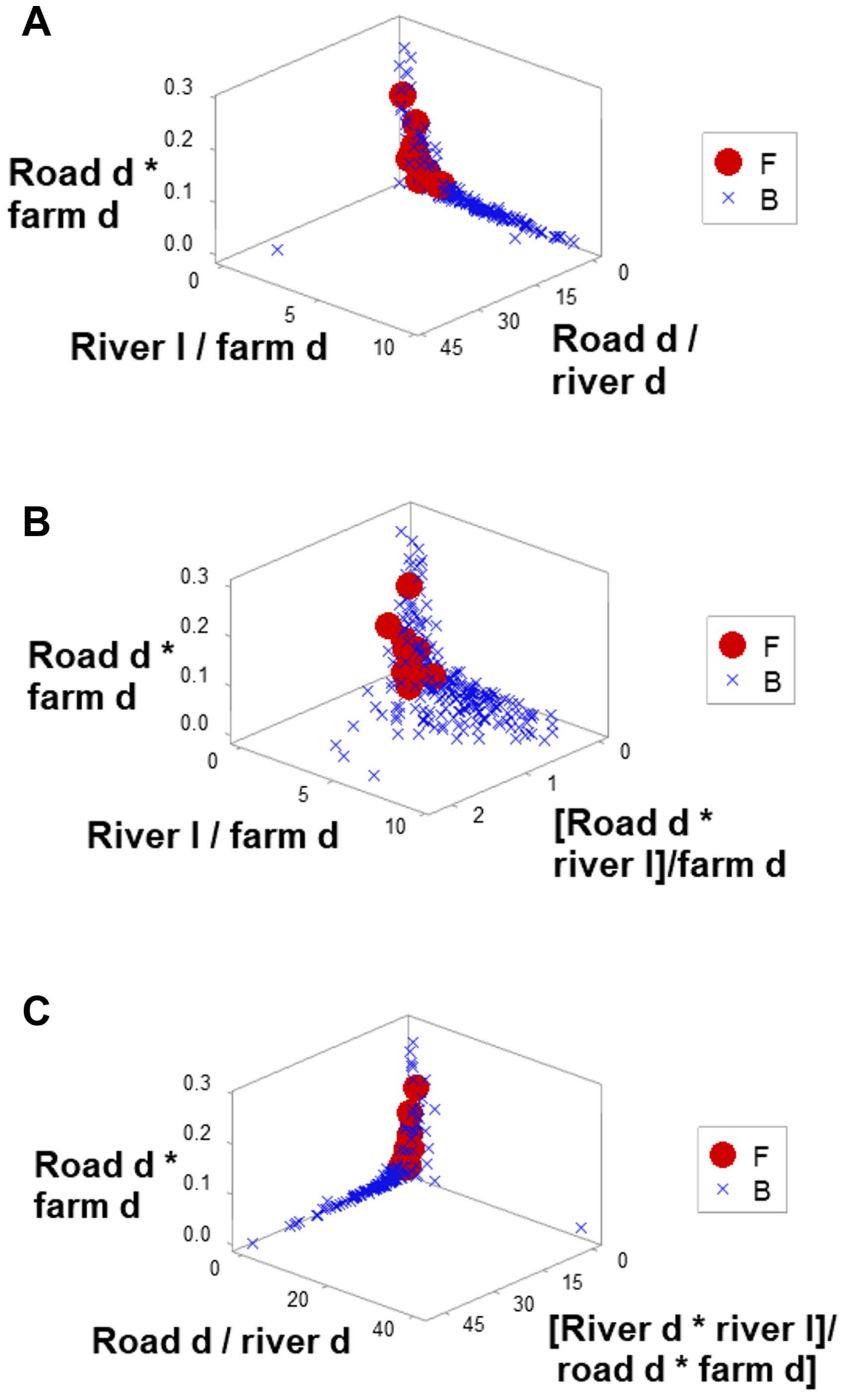
Three-dimensional analysis of geo-referenced, complex physical relationships that may influence disease dispersal. Three-dimensional analysis of complex indicators that only involved physical variables, such as the county-related length of rivers or county-related road density, displayed one or more perpendicular data inflections **(A–C)**. Such inflection differentiated two data subsets, of which one did not include counties affected by FMD (B counties). Because all variables interact in 3D space, it is assumed that the overall pattern captures, at least, seven interactions. Because spatial patterns can be assessed even in the absence of infections, if these distinct spatial patterns were shown to be repeatable, they could be considered in anticipatory testing.

Ambiguity was also observed: observations similar in all variables and values could have different meanings (green boxes; Figures 6A,B). To further explore complexity, geographic data were also analyzed without consideration of epidemic data. Using the identifiers that, in the temporal (11-week long) analysis characterized F counties (those that reported most of the infections), a county-centered analysis explored whether combinations of variables could reveal patterns that differentiated the same 11 counties from the remaining (B-like) 264 counties. Three levels of complexity were then evaluated: (i) complexity level I (bi-dimensional relationships between two variables; Figures 7A–C); (ii) complexity level II (bi-dimensional relationships between three variables; Figure 7D); and (iii) complexity level III [three-dimensional (3D) relationships among complex [more than three] variables; Figures 8A–C). Statistically significant differences were found between F and B-like counties when four indicators (complexity levels I and II) were assessed (all with *p* ≤ 0.01, Mann–Whitney test; Figures 7A–D). Three-dimensional analyses detected an additional emergent pattern: most B-like counties were orthogonal to F counties. While the vertical data subset included all F and some B-like counties, the horizontal subset only included B-like counties (Figures 8A–C).

The 3D analysis of physical structures distinguished two subsets that were perpendicular to one another (Figures 8A–C). If epidemiologic data were considered, the ‘vertical’ subset would include all F counties (and a few B counties) while the ‘horizontal’ subset would be 100% free of infections (Figures 8A–C). Because the orthogonal patterns were detected regardless of epidemiologic status, if these patterns were repeatable, any county included in the ‘horizontal’ subset could be suspected to become a barrier if an epizootic took place.

## Discussion

This study supported two novel inferences: (i) properties associated with complexity may be found when methods utilize geo-referenced and temporal data; and (ii) complex combinations of non-biological, geo-referenced data (such as the road and river networks) may reveal non-randomly distributed structures with potential influence on disease dispersal, which may be detected even in the absence of epidemiologic data. Methodological consequences associated with these inferences and some areas of possible applications are here discussed.

While unstructured data –observations on simple or isolated variables– were non-informative (Figure 4C), data structures that captured several levels of complexity described both a dynamic process (the epidemic) and a static (geographical) structure (Figures 4A,B, 8A–C). Findings support the view that epizootics reveal *emergence*, *irreducibility*, and *unpredictability* –properties typical of complex systems. The analysis of geo-referenced complexity may, at least partially, explain FMD outbreaks (21, 30, 31).

Two properties that may affect data analysis were also documented: data *circularity* and *spatial–temporal biological relativity* (32, 33). Such properties may prevent the use of models that analyze finite data intervals because circular data structures have no beginning and no end and, therefore, there are no explicit endpoints (34). As shown in Figures 6A,B, when relativity occurs, observations with similar numerical values may have different, if not opposite meaning (35). Yet, geo-referenced and dynamic analyses may distinguish such false similarities: the potential problems associated with ‘biological relativity theory’ and/or data circularity can be circumvented when complex properties are assessed with pattern recognition-oriented approaches. When an unambiguous pattern is determined, discrimination is possible (32, 35, 36).

Specifically, time-related arrows (data directionality) facilitated interpretation, even when circularity and relativity were observed. For example, two observations that showed similar numerical values were distinguished epidemiologically when temporal information (arrows that indicated where a data point was coming from/going to) were considered: one preceded the early epidemic phase, the other preceded the later phase (black open circle and blue closed square, respectively, Figure 4A).

Findings also indicated that, when data circularity is observed, no dichotomy is true (37). In contrast, non-binary models (those that consider there may be three or more epidemic stages) may prevent errors. As expected, complexity analysis extracted more or new information (20). Additional information was associated with data structures that captured many levels of complexity: level III indicators (which simultaneously captured 7 or more interactions, as shown in Figures 7A–D) yielded more information than simple (non-structured) variables (such as those reported in Figure 4C).

To discriminate, ‘top-down’ and ‘bottom-up’ aspects were considered (38). In particular, two challenges were addressed: (i) the need to structure the data in a way such that a complex host-microbial-geo-temporal system could be evaluated even without knowing, *a priori*, which data components would inform (a ‘top-down’-related problem); and (ii) the computational challenge associated with a very large number of data combinations to be analyzed (a potential problem associated with ‘bottom-up’ approaches). Both obstacles were overcome using an operation oriented to reveal distinct spatial patterns.

The adopted strategy prevented the ‘combinatorial explosion’ (39). This problem (also known as the ‘curse of dimensionality’ or ‘combinatorial complexity’) refers to analytical situations in which the number of possible combinations exceeds the number of variables and may approach infinity. For example, if 10 locations may experience 3 different events (to be disease-free, to be currently infected and within the exponential growth phase, or to be still infected but within the late or resolution phase), there are 3^10^ (~ 59,000) possible combinations. If the analysis of each of such combinations took 1 h, the whole analysis would require 6.7 years. While some numerical approaches have attempted to reduce the length of combinatorial analyses (40), other approaches have addressed the combinatorial explosion by focusing on spatial relationships (41). Because they tend to be more informative than one- or two-dimensional alternatives, this study followed the 3D approach.

Complex data structures demonstrated to be less variable than unstructured data (42, 43). Such features matter when validity is explored. This study investigated four dimensions of validity (44). *Construct validity* (detection of emergence, expressed as F and B counties) was shown at least eight times, as Figures 3 and 7 document. Because different data structures revealed emergence, *internal validity* was demonstrated. Because one physical geo-referenced pattern (a river acting as a barrier) has been reported in South African FMD epidemics (45), *external validity* was supported. Because statistical significance was documented at least four times (Figures 7A–D), *statistical validity* was demonstrated.

The notion that the *number* of ‘facilitators’ was not as relevant as their *geo-demographic location* and *structure* was supported: the observed geo-referenced network may be a part of a connecting network (28, 46). In several diseases, the geographical structure may determine whether disease dispersal occurs synchronically (47, 48). However, other factors (such as a ‘network of networks’) may also influence disease dissemination (49).

Findings may apply to human diseases, including human cholera and mosquito-borne diseases such as malaria and dengue (50). Climate-related factors –such as El Niño –induce ocean warming, which promotes long-distance dissemination of infectious agents (51–53). While the approach here explored is not necessarily applicable to epidemics of low morbidity, such as Ebola (54), it may apply in rapidly disseminating infectious diseases (46). These concepts also apply to wildlife surveillance and One Health approaches, where positive correlations have been reported between forest density and improved public health (55–59).

These considerations may improve interventions meant to stop epidemics. For example, practices that assume static situations and lack of interactions could be discontinued (60). They could be replaced with assumption-free, dynamic assessments of the local geography, which facilitate anticipatory allocation of resources and may lead to less costly and/or more effective control policies (46). Because complexity is associated with hidden interactions (61) and physical geo-referenced structures are independent of and/or precede epidemics, research on multiple geographic variables suspected to facilitate (or prevent) disease dispersal can uncover patterns usually unobserved. Hence, the analysis of geographical complexity is suggested. To that end, additional validations conducted in different bio-geographies are recommended.

## Data availability statement

The datasets presented in this study can be found in online repositories. The names of the repository/repositories and accession number(s) can be found in the article/Supplementary material.

## Author contributions

AH, JF, and MK: writing. AR and FF: methodology. SS: software. All authors contributed to the article and approved the submitted version.

## Conflict of interest

MK is employed by KB One Health LLC.

The remaining authors declare that the research was conducted in the absence of any commercial or financial relationships that could be construed as a potential conflict of interest.

## Publisher’s note

All claims expressed in this article are solely those of the authors and do not necessarily represent those of their affiliated organizations, or those of the publisher, the editors and the reviewers. Any product that may be evaluated in this article, or claim that may be made by its manufacturer, is not guaranteed or endorsed by the publisher.
