# Supplementary material for: Assessing complexity and dynamics in epidemics: geographical barriers and facilitators of foot-and-mouth disease dissemination
Source: Front Vet Sci. 2023 May 12;10:1149460. doi: 10.3389/fvets.2023.1149460 (PMC10213354; doi:10.3389/fvets.2023.1149460)
Supplement: Supplementary file 1 [file Data_Sheet_1.doc]

Supporting Materials

Table 1.

The data utilized in this study can be found at:

<https://cornell.app.box.com/s/oh7u5dgkhda0slyo05lxzs56c08mv3ac>

Supporting Materials

Table 2. Correlations between geographical variables, excluding FMD case data (n=275 counties)

| Correlation | *r* | *P* value | Epidemic time |
| --- | --- | --- | --- |
| Farm density vs. road density | 0.40 | <0.01 | Weeks 1-11 |
| Farm density vs. river density | -0.16 | <0.01 | Weeks 1-11 |
| Farm density vs. road length | 0.34 | <0.01 | Weeks 1-11 |
| Farm density vs. river length | 0.02 | >0.10 | Weeks 1-11 |
| Road density vs. river density | –0.38 | <0.01 | Weeks 1-11 |
| Road density vs. road length | 0.53 | <0.01 | Weeks 1-11 |
| Road density vs. river length | –0.06 | >0.10 | Weeks 1-11 |
| River density vs. road length | 0.35 | <0.01 | Weeks 1-11 |
| River density vs. river length | 0.19 | <0.01 | Weeks 1-11 |
| Road length vs. river length | –0.37 | <0.01 | Weeks 1-11 |

Supporting Materials

Table 3. Correlations between geographical variables, including FMD cases (n= 29 counties [week 1] or 71 counties [week 2]).

**A**

| Correlation | *r* | *P* value | Epidemic time |
| --- | --- | --- | --- |
| Cases vs. farm density | 0.33 | 0.08 | Week 1 |
| Cases vs. road density | 0.28 | >0.10 | Week 1 |
| Cases vs. river density | –0.26 | >0.10 | Week 1 |
| Cases vs. road length | –0.13 | >0.10 | Week 1 |
| Cases vs. river length | 0.11 | >0.10 | Week 1 |
|  |  |  |  |
| Farm density vs. road density | 0.52 | 0.04 | Week 1 |
| Farm density vs. river density | –0.03 | >0.10 | Week 1 |
| Farm density vs. road length | 0.44 | 0.02 | Week 1 |
| Farm density vs. river length | –0.13 | >0.10 | Week 1 |
|  |  |  |  |
| Road density vs. river density | 0.16 | >0.10 | Week 1 |
| Road density vs. road length | 0.55 | 0.02 | Week 1 |
| Road density vs. river length | 0.06 | >0.10 | Week 1 |
|  |  |  |  |
| River density vs. road length | 0.37 | 0.05 | Week 1 |
| River density vs. river length | –0.23 | >0.10 | Week 1 |
|  |  |  |  |
| Cases vs. farm density | 0.26 | 0.03 | Week 2 |
| Cases vs. road density | 0.30 | 0.01 | Week 2 |
| Cases vs. river density | –0.03 | >0.10 | Week 2 |
| Cases vs. road length | –0.10 | >0.10 | Week 2 |
| Cases vs. river length | 0.10 | >0.10 | Week 2 |
|  |  |  |  |
| Farm density vs. road density | 0.56 | <0.01 | Week 2 |
| Farm density vs. river density | –0.03 | >0.10 | Week 2 |
| Farm density vs. road length | 0.33 | <0.001 | Week 2 |
| Farm density vs. river length | –0.173 | >0.10 | Week 2 |
|  |  |  |  |
| Road density vs. river density | –0.19 | >0.10 | Week 2 |
| Road density vs. road length | 0.38 | <0.01 | Week 2 |
| Road density vs. river length | 0.00 | >0.10 | Week 2 |
|  |  |  |  |
| River density vs. road length | 0.05 | >0.10 | Week 2 |
| River density vs. river length | –0.32 | <0.01 | Week 2 |

Supporting Materials

Table 4. Epidemic dynamics in facilitator (F) and barrier (B) counties.*

A: Number of infected counties reporting new cases per epidemic week

| Epidemic  week | F  counties | B  counties | All  counties |
| --- | --- | --- | --- |
| 1 | 5 | 24 | 29 |
| 2 | 5 | 66 | 71 |
| 3 | 6 | 58 | 64 |
| 4 | 5 | 72 | 77 |
| 5 | 5 | 65 | 70 |
| 6 | 6 | 73 | 79 |
| 7 | 6 | 68 | 74 |
| 8 | 3 | 45 | 48 |
| 9 | 1 | 29 | 30 |
| 10 | 0 | 18 | 18 |
| 11 | 0 | 16 | 16 |

B: Cases, area, and case density of infected counties per epidemic week*

| Epidemic week | F  cases | B  cases | All cases | F area  (sq km) | B area  (sq km) | All areas  (sq km) | F case density | B case density | All case density |
| --- | --- | --- | --- | --- | --- | --- | --- | --- | --- |
| 1 | 12 | 71 | 83 | 3234.69 | 20599.6 | 23834.3 | 0.00338 | 0.00329 | 0.00336 |
| 2 | 22 | 180 | 202 | 2980.95 | 63937.9 | 66918.9 | 0.00492 | 0.00218 | 0.00230 |
| 3 | 19 | 176 | 195 | 4874.29 | 53901.5 | 58775.8 | 0.00492 | 0.00221 | 0.00237 |
| 4 | 23 | 249 | 272 | 3234.69 | 71053.7 | 74288.4 | 0.00656 | 0.00218 | 0.00230 |
| 5 | 15 | 242 | 257 | 3908.25 | 63354.7 | 67262.9 | 0.00246 | 0.00284 | 0.00274 |
| 6 | 21 | 195 | 216 | 4056.06 | 66187.6 | 70243.6 | 0.00543 | 0.00222 | 0.00237 |
| 7 | 9 | 149 | 158 | 2917.70 | 62321.3 | 65239.0 | 0.00417 | 0.00196 | 0.00202 |
| 8 | 8 | 78 | 86 | 1867.96 | 39020.7 | 40888.6 | 0.00338 | 0.00205 | 0.00218 |
| 9 | 1 | 38 | 39 | 821.37 | 25203.6 | 26025.0 | 0.00121 | 0.00143 | 0.00133 |
| 10 | 0 | 25 | 25 | 0 | 16561.3 | 16561.3 | 0 | 0.00143 | 0.00143 |
| 11 | 0 | 17 | 17 | 0 | 14429.6 | 14429.6 | 0 | 0.00123 | 0.00123 |
| Total | **130** | **1420** | **1550** |  | | | | | |

*The case density values here reported are the medians corresponding to a specific epidemic week. To that end, the values reported in the overall table (Table 1, Supporting Materials) were extracted on a per week basis and the medians for all observations corresponding to each (F, NF) group of counties were then calculated.
